# Combination Immune Checkpoint Blockade Strategies to Maximize Immune Response in Gynecological Cancers

**DOI:** 10.1007/s11912-018-0740-8

**Published:** 2018-11-13

**Authors:** Ying L. Liu, Dmitriy Zamarin

**Affiliations:** 1 Department of Medicine, Memorial Sloan Kettering Cancer Center, New York, NY USA; 2 Weill Cornell Medical College, New York, NY USA; 3 Gynecologic Medical Oncology, Immunotherapeutics Service, Memorial Sloan-Kettering Cancer Center, 300 East 66th street, 1313, New York, NY 10065 USA

**Keywords:** Ovarian cancer, Endometrial cancer, Cervical cancer, Immunotherapy, Checkpoint blockade

## Abstract

**Purpose of Review:**

Immune checkpoint blockade targeting PD-1 and PD-L1 improves immune recognition of tumor cells but had only modest success in gynecological cancers as monotherapy. Growing focus has been placed on combination immunotherapy strategies to overcome this resistance, and this review serves to discuss some of the most promising studies in gynecological cancers.

**Recent Findings:**

PD-1- and PD-L1-targeting antibodies are being combined with many novel agents including anti-CTLA-4 antibodies, PARP inhibitors, targeted agents, and traditional chemotherapy in promising studies with the hopes of increasing the immune response and overcoming resistance by targeting other pathways. Novel immune techniques including vaccines and adoptive cell therapies are also being implemented in gynecological cancers.

**Summary:**

Immune checkpoint combinations and novel immunotherapy strategies have demonstrated potential to overcome resistance to PD-1/PD-L1 blockade in gynecological cancers. Identification of biomarkers of response and resistance is a priority to tailor specific combination therapies to the appropriate patients.

## Introduction

The immune system is increasingly recognized as an important component of tumor detection and destruction. Recognition of tumor cells stimulates an immune cascade, resulting in T cell activation and migration and culminating in T cell-mediated destruction of tumor cells. However, this process requires a number of steps and co-stimulatory signals, and cancers have developed many mechanisms of resistance to evade detection by the immune system [1].

Activation of tumor-specific T cells requires binding of the T cell receptor antigen peptide presented by the major histocompatibility complex (MHC) class I and II molecules on the surface of antigen-presenting cells (APCs). This process requires activation of a co-stimulatory receptor such as CD28 or B7 on the T cell surface. T cells also express a wide variety of other co-stimulatory and inhibitory receptors, which can affect this complex process [2, 3].

Much of immunotherapy thus far has focused on targeting of the inhibitory receptors on the surface of T cells and their ligands, a strategy that has been termed immune checkpoint blockade. In particular, blocking the programmed death receptor 1 (PD-1) and its ligand PD-L1 has been shown to reverse and/or prevent tumor-associated T cell exhaustion, promoting the activation of tumor detection and destruction.

Although effective in many other tumor types, the use of checkpoint inhibitors as monotherapy has only had moderate success in gynecological cancers. Findings from various cancer types highlight that mechanisms underlying the tumor immune response are extremely complex and involve many different aspects of the host immune system, tumor microenvironment, tumor genomics, and cytokine/vascular milieu [4].

**Fig. 1:**
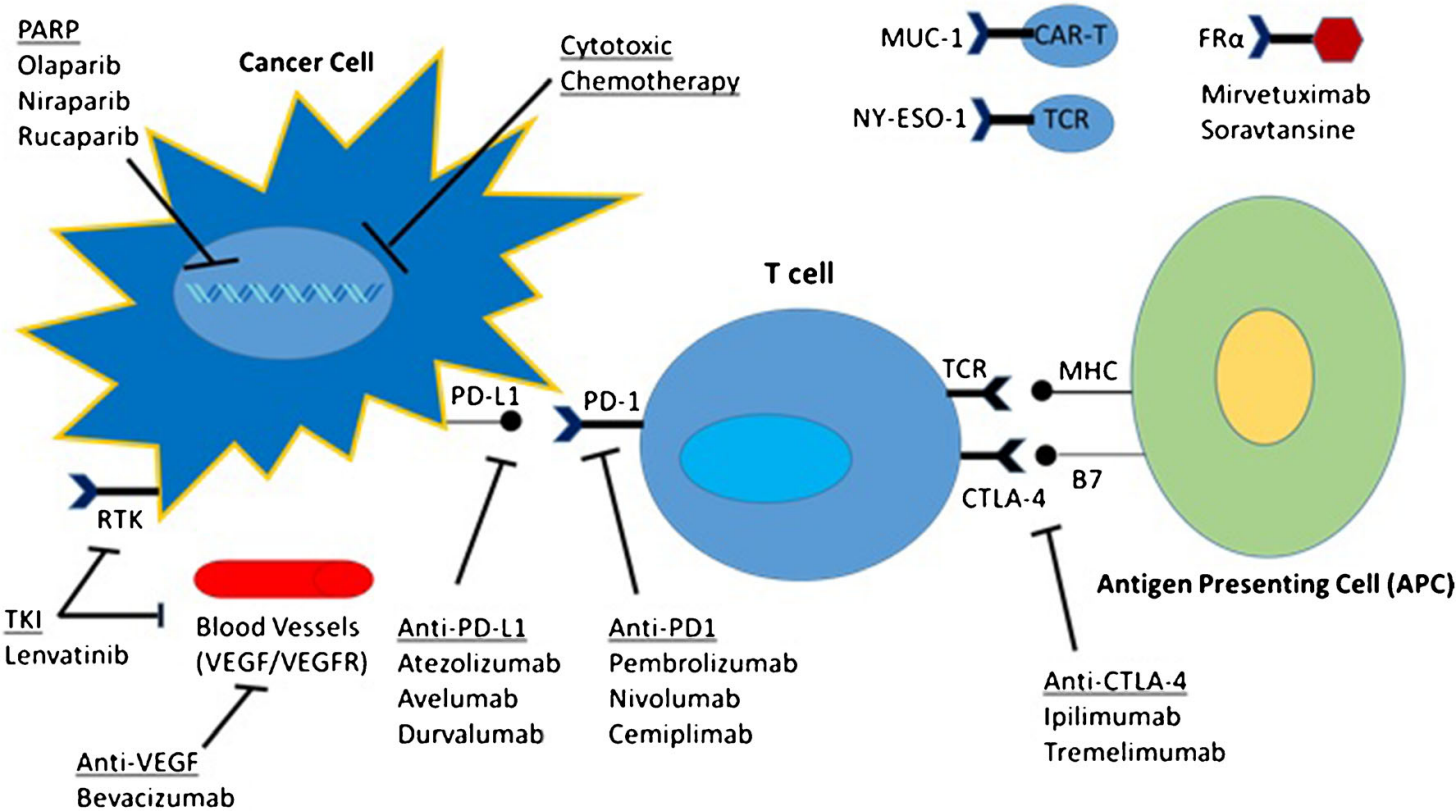
Mechanisms of immunotherapy combinations in gynecological cancers. Legend: Novel studies in gynecological cancers are combining checkpoint blockade with multiple therapies including traditional chemotherapy, PARP inhibitors, anti-VEGF agents, and anti-CTLA-4 antibodies, potentially targeting multiple mechanisms and overcoming resistance. Abbreviations: TCR T cell receptor, TKI tyrosine kinase inhibitor, RTK receptor tyrosine kinase, FRα folate receptor alpha

As a result, more studies are focusing on therapies targeting these other pathways in combination with checkpoint inhibitors in order to enhance their efficacy and overcome resistance [5]. Figure 1 depicts the various mechanisms that are being investigated in gynecological malignancies. This review serves to highlight the most promising studies to date using combination immunotherapy in various areas of gynecological malignancies.

## Ovarian Cancer

There are approximately 22,000 new cases of ovarian cancer diagnosed each year in the USA. Of these, over 70% are diagnosed with advanced-stage disease (Stage III/IV), and despite improving treatments, 5-year survival is only 47% (SEER Stat Fact sheets, accessed 7/2018).

### Checkpoint Inhibitor Monotherapy

Although promising in other cancer types, single-agent checkpoint inhibition has only produced modest results in ovarian cancers [6]. In a phase II trial of nivolumab (anti-PD-1) monotherapy in 20 patients with platinum-resistant ovarian cancer, the best overall response rate (ORR) was 15% with disease control rate (DCR) of 45%. Median progression-free (PFS) and overall survival (OS) were 3.5 and 20 months, respectively [7•]. In a phase II study of pembrolizumab (anti-PD-1) in 376 women with platinum-resistant ovarian cancer (1–6 prior lines), the ORR was 8.0% (7 CRs, 23 PRs) with DCR of 37.2%, but response may be higher in those with high PD-L1 expression (18% ORR in those with >10% expression) [8•]. In a phase IB study of avelumab (anti-PD-L1) in 124 patients with refractory/resistant ovarian cancer, the ORR was 9.7% with a DCR of 54%. Median PFS was 11.3 weeks, and mOS was 10.8 months [9•]. Given low ORRs of 10–15% with checkpoint blockade monotherapy in refractory ovarian cancer, many studies have focused on combination therapies to improve response rates.

### Combination Checkpoint and CTLA-4 Inhibition

Cytotoxic T cell antigen-4 (CTLA-4) is another inhibitory checkpoint involved in T cell priming and activation [1], and translational studies in ovarian cancer have demonstrated that dual blockade of PD1/PDL1 and CTLA-4 increases tumor-infiltrating lymphocyte (TIL) activation and antigen-specific inflammatory cytokine production and decreases Tregs, potentially facilitating tumor rejection [10]. CTLA-4 blockade may also be potentiated by prior vaccination. In a group of 11 women treated with the GVAX vaccine (autologous tumor cells engineered to secrete GM-CSF) followed by ipilimumab (anti-CTLA-4), one patient had a dramatic fall in her CA-125 levels and 3 other patients achieved stabilization of their disease [11]. Combination of CTLA-4 blockade with PD-1 blockade has demonstrated efficacy in various cancer types, including melanoma, bladder, kidney, and lung cancer [12–16]. Given these promising results, the NRG Oncology Group has conducted a phase II study in 100 patients with platinum-resistant ovarian cancer, comparing combination nivolumab/ipilimumab to nivolumab alone in relapsed ovarian cancer within 12 months of last platinum therapy (NCT02498600). The study has completed accrual, and the results are expected toward the end of 2018.

### Immunotherapy and Chemotherapy

Checkpoint blockade is also being studied in combination with traditional chemotherapy in many different areas of ovarian cancer. Preclinical data suggest that chemotherapy may enhance the immune response to checkpoint blockade through multiple mechanisms including inhibiting the immunosuppressive machinery of tumor cells, increasing tumor antigen exposure and DNA damage, and facilitating penetration of immunotherapy agents [17, 18]. In lung cancer, recent phase III trials of upfront platinum-based chemotherapy in combination with pembrolizumab in lung adenocarcinoma and lung squamous cell carcinoma have demonstrated marked survival benefit of the combinations, when compared to chemotherapy alone [19•]. These findings generate a high rationale for evaluation of PD-1/PD-L1 blocking agents in combination with chemotherapy in epithelial ovarian cancer, and several studies are ongoing, as outlined below.

### Upfront Therapy and Platinum-Sensitive Recurrence

In the upfront setting, the JAVELIN study is examining the role of the PD-L1 antibody avelumab added to carboplatin and paclitaxel (NCT02718417). It is an international, open-label phase III study with three arms: carboplatin/paclitaxel, carboplatin/paclitaxel followed by avelumab maintenance, or carboplatin/paclitaxel/avelumab followed by avelumab maintenance.

There is increasing evidence that targeting vascular endothelial growth factor (VEGF) in combination with immune checkpoint inhibition can enhance therapeutic efficacy [20, 21], generating a rationale for incorporation of such strategies into immunotherapy combinations in ovarian cancer. IMAGYN50 is a randomized multicenter phase III trial examining the role of the PD-L1 antibody atezolizumab in combination with carboplatin, paclitaxel, and bevacizumab followed by bevacizumab and atezolizumab maintenance vs. placebo in the upfront setting (NCT03038100).

This same regimen is also being investigated after first or second platinum sensitive recurrence (> 6 months since last platinum therapy) in the ATALANTE trial (ENGOT-ov29), which is an international randomized phase III study whose design was presented at ASCO 2018 (NCT02891824) [22]. The studies will also evaluate the role of immune checkpoint blockade in maintenance in order to maximize the potential durability of such combinations.

### Platinum-Resistant Recurrence

The combination of checkpoint blockade and chemotherapy has also shown promise in the platinum-resistant setting. Matulonis et al. presented the preliminary results of a phase II study of pegylated liposomal doxorubicin (PLD) combined with pembrolizumab in 26 women with platinum-resistant ovarian cancer at SGO 2018. The ORR was 19%, 5 partial responses (PR’s), with a DCR at 6 months of 42%, with 5 PRs and 6 stable disease (SD). The combination was reasonably well tolerated with grade 3–4 adverse events (AE’s) of anemia (12%), rash (12%), and increased liver enzymes (12%) [23].

Two other studies are investigating PLD and immunotherapy combinations. JAVELIN Ovarian 200 is a phase III study randomizing women with platinum resistant or refractory platinum sensitive (< 3 lines of therapy) to one of three arms: PLD plus avelumab, avelumab alone, or PLD alone with primary endpoints of PFS and OS [24]. PLD is also being investigated with durvalumab in a phase I/II study (NCT02580058) conducted in women with platinum-resistant ovarian cancer. The study has finished accrual, and results will be presented at ESMO 2018.

The combination of chemotherapy with anti-VEGF therapy and checkpoint inhibition is also being investigated in the platinum-resistant setting. NCT03353831 is a phase III, multicenter, randomized trial of chemotherapy (PLD or weekly paclitaxel) with bevacizumab plus atezolizumab vs. placebo in women with first or second relapse within 6 months of last platinum therapy or third relapse. The NRG Oncology Group is also conducting a phase II/III trial in women with platinum-resistant ovarian cancer that randomizes them to PLD/atezolizumab/bevacizumab vs. PLD/atezolizumab vs. PLD/bevacizumab (NCT02839707). The results of these trials are pending but have the potential to change practice by incorporating checkpoint blockade into standard therapy at multiple time points in ovarian cancer.

### Immunotherapy and PARP Inhibitors

DNA damage and genomic instability may drive immune response, and several studies are investigating combining checkpoint inhibitors with agents targeting DNA damage pathways in hopes of improving response rates [25, 26].

The PARP (poly ADP-ribose) family of enzymes are essential for repairing single-stranded DNA breaks. PARP inhibitors effectively trap PARP and induce apoptosis in cells harboring defects in their homologous recombination (HR) or double-stranded DNA repair functions, commonly seen in BRCA1/2 mutated tumors, causing a “synthetic lethality” [27]. Although approved as single agents in breast and ovarian cancers, mostly in those with BRCA 1/2 mutations, many studies are investigating the combination of PARP inhibitors with immune checkpoint blockade in all-comers.

In a phase I study in women’s cancers (ovarian, triple negative breast (TNBC), cervical and uterine), Lee et al. evaluated the anti-PD-L1 antibody durvalumab in combination with olaparib in 12 patients (10 had ovarian cancer and 2 had TNBC), 11 of which were BRCA wild type (BRCAwt) and 1 was unknown. The combination was well tolerated, and two women achieved PRs and 8 women had SD, achieving an 83% DCR [28].

Friedlander et al. presented the results of a phase Ib basket study of the anti-PD-1 monoclonal antibody (BGB-A317) in combination with PARP inhibitor (BGB-290) in advanced solid tumors (BRCA mutation unspecified) at ASCO 2017. In the 38 patients treated, 7 achieved a PR (5 with ovarian cancer and 1 with uterine cancer), and 1 woman with ovarian cancer achieved a CR. A dose expansion in multiple tumor types is in progress [29].

Drew et al. reported the interim results of a phase II basket study (MEDIOLA) of durvalumab plus olaparib in 32 patients with platinum-sensitive relapsed ovarian cancer and germline BRCA 1/2 mutations. The combination resulted in an ORR of 63% (6 CRs and 14 PRs) and overall DCR at 12 weeks of 81%. The regimen was well tolerated with grade 3–4 AEs of anemia (9%), increased lipase/amylase (6–9%), and neutropenia (3%) [30].

The TOPACIO trial is a phase I/II study of pembrolizumab plus niraparib in women with both BRCA mutated and wild-type (WT) ovarian cancers and TNBC. The interim results in the 36 patients with heavily pre-treated, platinum-resistant ovarian cancer found an ORR of 27% (6 PRs, 3 were BRCAwt) and a DCR of 50%. The combination was also well-tolerated with grade 3–4 AEs of anemia (17%), fatigue (6%), and thrombocytopenia (3%) [31•].

Finally, the ATHENA trial is a phase III, randomized trial investigating rucaparib and nivolumab as maintenance following response to upfront platinum-based therapy in stage III/IV ovarian cancer (NCT03522246). The four arms will include maintenance nivolumab plus rucaparib, nivolumab plus placebo, rucaparib plus placebo, or placebo alone. The study is ongoing with plans to analyze the results stratified by homologous repair deficiency (HRD) status.

Combination PARP and immunotherapy represents one of the most promising areas in ovarian cancer with impressive response rates in both BRCA 1/2 mutated and WT patients. These studies also provide opportunities for translational studies to help elucidate additional mechanisms of DNA damage pathways and resistance to PARP inhibitors.

### Adoptive Cell Therapies

Adoptive cell therapies range from utilizing autologous tumor infiltrating lymphocytes (TILs) to implementing engineered exogenous T cell receptors (TCRs) or chimeric antigen receptors (CARs) to combat tumor cells. Some of these therapies have been approved in hematological malignancies; however, their use in solid tumors is still limited [32]. One of the earliest studies conducted in ovarian cancer involved treatment of 13 patients with adoptive transfer of TILs, which resulted in 3-year disease-free survival rate of 82.1% and OS of 100% [33]. Since then, several studies have examined the efficacy of adoptive cell therapies in this population.

CAR-T therapy targeting extracellular domains of MUC16, an antigen expressed on most ovarian carcinomas, have been studied in preclinical models and have shown the potential to delay progression or fully eradicate the disease in mouse models [34, 35]. This work was translated into a phase I clinical trial of MUC-16 directed CARs in women with recurrent ovarian cancer that is currently ongoing (NCT02587689) [36].

NY-ESO-1 is a cancer germline antigen expressed in many solid tumors, including ovarian cancers, but not normal tissues with the exception of the immunologically privileged sites such as gonads [32]. NY-ESO-1 has also been associated with an aggressive phenotype of ovarian cancer [37], making it an attractive antigen for TCR therapies. Two phase I/IIb studies are currently ongoing in patients with recurrent ovarian cancer to evaluate the safety and efficacy of NY-ESO-1 targeted TCR therapy (NCT01567891 and NCT02650986).

Although the area of adoptive cell therapies is promising, this work is still early in ovarian cancer. The process of administration of these therapies is also intensive and requires identification of patients whose tumors express the appropriate targets as well as HLA matching for patients receiving engineered TCR therapies.

### Vaccine Strategies

As ovarian cancer is an immunogenic cancer, vaccine strategies utilizing different targets and platforms have also been tested and described in detail elsewhere [38, 39]. One of the shared vaccine targets commonly expressed in ovarian cancer is the folate receptor, which has been a target for both antibody-based therapies and vaccines [40]. Mirvetuximab soravtansine is an antibody-drug conjugate that targets the folate receptor α (FRα) and is coupled with a cytotoxic effector compound (maytansinoid DM4). In phase I studies it has been shown to be tolerable with a signal of response (ORR 47% and PFS of 6.7 months) [41]. Matulonis et al. presented a phase Ib study of mirvetuximab soravtansine with pembrolizumab in 14 women with heavily pre-treated ovarian cancer with an ORR of 43% and PFS of 5.2 weeks [42].

TPIV200 is a vaccine targeted toward FRα, and in a phase I study including 14 women with ovarian cancer, the vaccine was safe and elicited or augmented immunity [43]. As a result, there is an ongoing study comparing TPIV200 with GM-CSF vs. GM-CSF alone as a maintenance therapy after upfront chemotherapy in ovarian cancer (NCT02978222). Vaccines are also being combined with checkpoint blockade to potentially enhance activity. In a phase II study of 27 women with recurrent platinum-resistant ovarian cancer presented at SGO 2018, the combination of TPIV200 with GM-CSF and durvalumab was safe and tolerable and resulted in durable disease control in a number of patients [44]. O’Cearbhaill et al. also reported the results of a peptide vaccine targeting WT1, which is highly expressed on serous ovarian cancer, combined with nivolumab in 11 women with platinum-sensitive recurrent ovarian cancer given as maintenance to the patients who achieved a complete remission following platinum-based chemotherapy. The combination was well-tolerated, elicited antigen-specific T cell responses, and resulted in a 1-year PFS rate of 64% [45].

## Endometrial Cancer

Endometrial cancer is the most common gynecological cancer in the USA with over 60,000 newly diagnosed cases and almost 12,000 deaths each year [46]. It has become clear through efforts such as the cancer genome atlas project (TCGA) [47] that endometrial cancers are a heterogeneous group of diseases that can be divided into four categories based on genomic characteristics: *POLE* ultramutated, microsatellite instability (MSI) hypermutated, copy number-low, and copy number-high.

### Checkpoint Inhibitor Monotherapy

Based on TCGA datasets, MSI is present in 30–40% of endometrioid endometrial cancers [47] and has been demonstrated to be a marker for response to anti-PD-1/PD-L1 antibodies [48, 49]. In a basket trial of 86 patients with mismatch repair (MMR)-deficient cancers and disease progression after prior therapy who were treated with pembrolizumab, there was an ORR of 53% with DCR of 73% in the 15 patients with endometrial cancer [50•]. In addition, the POLE-ultramutated subtype of endometrial cancer has the highest mutational burden and is hypothesized to respond well to checkpoint blockade [51, 52]. Subgroup analyses of trials of both pembrolizumab and nivolumab in endometrial cancers have shown durable clinical responses in MSI and POLE-mutated subtypes [53, 54].

Although successful in the MSI-H subtype of endometrial cancer, checkpoint blockade monotherapy in those with microsatellite stability (MSS) has proven less effective. In a phase Ia study of 15 women with recurrent endometrial cancer, atezolizumab every 3 weeks resulted in an ORR of 13% (2 PRs) and a DCR of 27%. Most of the women in the cohort were MSS, and of the two responders, one had MSI-H disease and the other had MSS disease but PD-L1 expression > 5% with disease heavily infiltrated with TILs [55].

In a phase Ib (KEYNOTE-028) study of pembrolizumab in advanced solid tumors, the 24 patients with recurrent metastatic endometrial cancer had an ORR of 13% (3PRs) and a DCR of 25% (3PRs and 3SD). The safety profile was acceptable, and the clinical benefit will be further investigated in the phase II KEYNOTE-158 trial [56•].

A phase II study evaluated avelumab in 31 patients with heavily pre-treated, recurrent endometrial cancer. Sixteen of the patients were MSS, and 15 were MSI/POLE mutated. In the MSS cohort, only one patient met criteria for PFS6 by iRECIST but not RECIST; thus, the study will not move on in this population. However, in the MSI/POLE cohort, the ORR was 20% (3PRs), and DCR was 33% (5 patients met PFS6). Avelumab was well-tolerated and will move into further study in the MSI/POLE cohort only [57].

### Combination Therapies

There is also growing interest in combining immunotherapy with both targeted agents, other immunotherapies, and chemotherapy in all endometrial cancer subtypes.

Makker et al. recently presented the results of a phase IB/II study of 54 patients (80% were MMR proficient) with recurrent endometrial cancer (majority with endometrioid histology) who were treated with a combination of pembrolizumab and lenvatinib, a small-molecule multi-kinase inhibitor targeting VEGF [58]. The ORR at 24 weeks was 50% in MSI-H and MSS patients. Median PFS was 10.1 months. The regimen was well tolerated with grade 3 AEs occurring in 58% of patients, most commonly hypertension (59%), fatigue (50%), diarrhea (44%), hypothyroidism (35%), and stomatitis (33%) [59•]. Given these promising results, the combination will be studied in a large, randomized, international phase III trial and has been granted breakthrough designation by the FDA.

Studies are also exploring the combination of chemotherapy with immunotherapy in endometrial cancer. There is an ongoing phase II trial examining carboplatin/paclitaxel plus pembrolizumab in advanced or recurrent endometrial cancer that is currently recruiting (NCT02549209). NRG-GY018 is a randomized phase III study of carboplatin/paclitaxel with or without pembrolizumab followed by maintenance pembrolizumab or placebo for measurable stage III/IV or recurrent endometrial cancer. The study will enroll patients regardless of MMR status but will stratify by MSI-H and MSS.

There is also an ongoing phase II study of combination PD-L1 and anti-CTLA-4 (durvalumab plus tremelimumab vs. durvalumab alone) in women with recurrent endometrial cancer regardless of MMR status (NCT03015129) that is currently enrolling.

Overall, while single-agent immune checkpoint blockade has been successful in a subset of MSI-H and POLE endometrial cancers, combinatorial strategies will be necessary to overcome resistance to immunotherapy in the majority of endometrial cancers.

## Cervical Cancer

There are more than 13,000 cases of cervical cancer with over 4000 deaths each year in the USA. Although treatments have improved for early-stage cervical cancer, overall survival is still poor in those with advanced disease (SEER Stat Fact sheets, accessed 7/2018).

The majority of cervical cancers are caused by human papillomavirus (HPV) infection, with HPV types 16 and 18 accounting for most cases. The virus infects epithelial cells and encodes two proteins (E6 and E7), which promote cellular proliferation, prolong cell-cycle progression, prevent apoptosis, and are essential to growth. The virus is cleared spontaneously in a vast majority of women by the host immune system, but it persists in some women by evading the immune system and causing cancer [60].

As a predominantly virally mediated cancer, cervical cancer may be uniquely amenable to immunotherapy techniques. As a result, multiple novel immunotherapy mechanisms and combination are being tested. A high proportion of cervical cancers also express PD-L1, which may have a prognostic and predictive role in treatment. As a result, there is particular interest in utilizing immunotherapy to facilitate host recognition of cervical cancers [61, 62].

### Checkpoint Inhibitor Monotherapy

Nivolumab monotherapy was studied in a phase I/II study in women with recurrent or metastatic cervical, vaginal, and vulvar cancers (CheckMate 358), and the results were presented at ASCO 2017. Of the 24 patients treated, the ORR was 20.8% with DCR of 70.8%, and all responses were in those with cervical cancer (*n* = 19). Responses were observed regardless of PD-L1 or HPV status [63]. Pembrolizumab monotherapy was studied in a phase Ib KEYNOTE-028 study and then in the larger expansion cohort (KEYNOTE-158) study in women with recurrent cervical cancer. Among the 71 total women treated, the ORR was 17%, [64, 65•] resulting in the recent approval of pembrolizumab in PD-L1 positive cervical cancer after progression on chemotherapy.

Immunotherapy is also being compared to chemotherapy in this setting. GOG3016/ENGOT-Cervix 9 is a phase III trial of cemiplimab (Anti-PD1) vs. investigator’s choice chemotherapy in recurrent/metastatic cervical cancer resistant to platinum-based therapy after two or more lines of treatment (NCT03257267) [66].

CTLA-4 blockade as monotherapy has also been evaluated in cervical cancer. A phase I/II study of ipilimumab in 42 patients with recurrent/metastatic cervical cancer demonstrated response in 1 patient and 10 patients with stable disease in the 34 evaluable patients. Grade 3 toxicities of diarrhea (*n* = 4) and colitis (*n* = 3) occurred infrequently [67].

### Combination Therapies

There is evidence of a potentially synergistic effect between radiation and the host immune response. The abscopal effect occurs when targeted radiotherapy to a specific tumor lesion can elicit an immune-mediated response in a non-targeted lesion [1, 68]. This phenomenon has been seen in metastatic non-small cell lung cancer (NSCLC) and in melanoma with concurrent radiotherapy plus ipilimumab [69, 70], but it has not been well studied in gynecological malignancies.

GOG 9929 is a phase I trial of ipilimumab for primary treatment of cervical cancer after chemoradiation. In the 20 evaluable patients, the 1 year DFS was 74% (compared to 55% in historical SEER databases). The therapy was well tolerated with only three patients having grade 3 toxicities, which were self-limiting [71].

Given these promising results, there is an upcoming randomized, phase II study of atezolizumab as an immune primer when given concurrently with extended field chemoradiotherapy for node-positive, locally advanced cervical cancer (NRG GY017). Similarly, there is an ongoing PAPAYA phase I trial of concurrent pembrolizumab with radiotherapy and cisplatin with newly diagnosed stage IB-IVA cervical cancer (NCT03144466). Atezolizumab is also being combined with bevacizumab in a phase II study in recurrent, persistent, or metastatic cervical cancer (NCT02921269).

### Vaccines and Adoptive Cell Therapies

The presence of viral antigens in cervical cancers provides for an opportunity to evaluate additional immunotherapeutic strategies.

Vaccine strategies to help boost the immune response to cervical cancer are being investigated in early and advanced/recurrent cervical cancers. VGX-3100 is a synthetic plasmid targeting HPV-16/HPV-18 E6 and E7 proteins. A phase IIb randomized, placebo-controlled trial studied VGX-3100 in 167 patients with HPV-associated cervical intraepithelial neoplasia (CIN) 2/3. In the modified intention-to-treat analysis 48.2% (55/114) of VGX-3100 recipients and 30% (12/40) of placebo recipients had histopathological regression (difference of 18.2%, *p* = 0.034). The vaccine was well-tolerated and showed promise as a non-surgical therapeutic option for CIN 2/3 [72].

ADSX 11-001 is a live-attenuated *Listeria monocytogenes* vaccine engineered to an antigen-adjuvant fusion protein consisting of a truncated fragment of listerolysin fused to human HPV-16 E7 protein. Upon administration, this fusion protein is taken up by antigen-presenting cells to activate the MHC-1 pathway. This vaccine was shown to be effective in reducing in vivo tumor burden in preclinical animal studies [73].

ADSX 11-001 was studied in patients with cervical cancer in two studies. The first was phase II randomized study of ADSX 11-001 with or without cisplatin. The mOS was comparable between the two arms (8.28 in the vaccine only group vs. 8.78 in the vaccine/cisplatin group). Rates of 12 and 18-month OS were similar between the two groups, and the therapies were well tolerated, although more AEs were reported in the combination group [74]. Given these findings, ADSX 11-001 was evaluated in the GOG 265 study in 50 women with recurrent/metastatic cervical cancer who had progressed after at least one line of chemotherapy. The study demonstrated a 12-month OS rate of 38.5%, which compared favorably to the historical 24% rate in this population [75].

Adoptive cell strategies are also being implemented in cervical cancer to boost the immune response. Nine patients with previously treated platinum-based chemotherapy or chemoradiotherapy (CRT) received a single infusion of TILs selected for HPV E6/E7 reactivity along with aldesleukin (IL-2). The ORR was 33% (2 CR, 1PR) with durable responses. There were no autoimmune toxicities, and observed toxicities were mostly hematological and related to the conditioning regimen [76]. A phase II study evaluating engineered TCR targeting HPV E7 is ongoing (NCT02858310).

### Future Directions/Conclusions

Although checkpoint blockade monotherapy has only resulted in modest advances in gynecological cancers thus far, promising combination strategies with other immunotherapy agents, targeted agents, chemotherapy, and radiation are currently under investigation (Tables 1 and 2). Novel immune therapies, such as vaccine strategies and adoptive T cell technologies, are also demonstrating promising preliminary results and will be tested in combination with immune checkpoint inhibitors in the future. Aside from the PD-L1 status and MSI status, identification of additional pre-treatment and on-treatment predictive biomarkers will be essential to improve patient selection and to identify the patients appropriate for the specific combinations. Finally, given the durability of responses seen with immunotherapy, optimal duration of therapy and the role of maintenance will also need to be addressed.

**Table 1:** Reported studies evaluating checkpoint inhibitor monotherapy and combination therapy in gynecological cancers

Treatment	Study design	Number	ORR	DCR	Reference
*Ovarian*					
Nivolumab	Phase II	20	15%	45%	Hamanishi et al. [6]
Pembrolizumab	Phase II	376	8% (7 CRs, 23PRs)	37.2%	Matulonis et al. ASCO [8]
Avelumab	Phase Ib	124	9.7%	54%	Disis et al. ASCO [9]
Pembrolizumab + PLD	Phase II	26	19% (5 PRs)	42% (5PR and 6 SD) at 6 mo	Matulonis et al. SGO [23]
Durvalumab + Olaparib	Phase II (BRCA 1/2 WT)	12	17% (2PRs)	83% (2PR and 8 SD)	Lee et al. [28]
BGB-A317 + BGB-290 (PD-1 and PARP)	Phase Ib	38	21% (1CR, 7PR’s)	not reported	Friedlander et al. ASCO [29]
Durvalumab + Olaparib (MEDIOLA)	Phase II (gBRCA 1/2 mutations)	32	63% (6 CRs and 14 PRs)	81% at 12 wks	Drew et al. SGO [30]
Pembrolizumab + Niraparib (TOPACIO)	Phase II (BRCA 1/2 WT and mutants)	36	27% (6 PRs)	50%	Konstantinopoulos et al. SGO [31•]
Mirvetuximab soravtansine + pembrolizumab	Phase Ib	14	43%	not reported	Matulonis et al. SGO [42]
*Cervical*					
Pembrolizumab	Phase Ib/II	71	17%	not reported	Schellens et al. ASCO [65•]
Nivolumab	Phase I/II	24	20.8%	70.8%	Hollebecque et al. ASCO [63]
Ipilimumab	Phase I/II	34	3% (1PR)	32%	Lheureux et al. ASCO [67]
*Endometrial*					
Pembrolizumab	Phase I/II basket (MSI-H)	15	53%	73%	Le et al. [50•]
Pembrolizumab	Phase I basket (MSI-H and MSS)	24	13% (3PRs)	25% (3PRs and 3SD)	Ott et al. ASCO [56•]
Atezolizumab	Phase Ia (MSI-H and MSS)	15	13% (2 PRs)	27%	Fleming et al. ASCO [55]
Avelumab	Phase II (MSI-H and MSS)	31	20% (3PRs) MSI-H only	33% (MSI-H only)	Konstantinopoulos et al. SGO [57]
Pembrolizumab + Lenvantinib	Phase Ib/II (80% MSS)	54	50% (24 weeks)	not reported	Makker et al. ASCO [59•]

*PLD* pegylated liposomal doxorubicin, *ORR* overall response rate, *DCR* disease control rate, *PR* partial response, *SD* stable disease, *CR* complete response, *MSI-H* microsatellite instability high, *MSS* microsatellite stable

**Table 2:** Ongoing combination immunotherapy trials in gynecological cancers

Treatment	Mechanism	Phase	Trial ID	Status
Ovarian				
*Upfront*				
Carboplatin/paclitaxel with or without avelumab (JAVELIN)	Chemo/PD-L1	III	NCT02718417	Open/not recruiting
Carboplatin/paclitaxel/bevacizumab/atezolizumab vs. placebo (IMAGYN50)	Chemo/VEGF/PD-L1	III	NCT03038100	Open/recruiting
Maintenance rucaparib + nivolumab vs. rucaparib vs. nivolumab vs. placebo (ATHENA)	PARP/PD1 following chemo	III	NCT03522246	Open/recruiting
*Relapsed/refractory*				
Carboplatin/paclitaxel/bevacizumab/atezolizumab vs. placebo (ATALANTE)	Chemo/VEGF/PD-L1	III	NCT02891824	Open/recruiting
Nivolumab/ipilimumab vs. nivolumab (NRG)	PD1/CTLA-4	II	NCT02498600	Open/not recruiting
PLD + avelumab vs. avelumab vs. PLD (JAVELIN Ovarian 200)	Chemo/PD-L1	III	NCT02580058	Open/not recruiting
PLD + durvalumab	Chemo/PD-L1	I/II	NCT02431559	Open/not recruiting
Chemo/bevacizumab/atezolizumab vs. placebo	Chemo/VEGF/PD-L1	III	NCT03353831	Not yet recruiting
PLD/bevacizumab/atezolizumab vs. PLD/atezolizumab vs. PLD/bevacizumab	Chemo/VEGF/PD-L1	II/III	NCT02839707	Open/recruiting
Cervical				
Pembrolizumab/RT/cisplatin (PAPAYA)	PD1/Chemo/RT	I	NCT03144466	Open/recruiting
Atezolizumab + CRT (NRG GY017)	PD-L1/Chemo/RT	III	pending	Pending
Atezolizumab + bevacizumab	PD-L1/VEGF	II	NCT02921269	Open, not recruiting
Endometrial				
Carboplatin/paclitaxel/pembrolizumab	Chemo/PD1	II	NCT02549209	Open/recruiting
Carboplatin/paclitaxel/pembrolizumab vs. placebo (NRG-GY018)	Chemo/PD1	III	pending	Pending
Durvalumab + tremelimumab vs. durvalumab	PD-L1/CTLA-4	II	NCT03015129	Open/recruiting

*PD1* programmed death ligand 1, *CTLA-4* CTL antigen-4, *PLD* pegylated liposomal doxorubicin, *CRT* chemoradiotherapy
